# The chromosomal genome sequence of the photosymbiotic ascidian, *Trididemnum clinides *Kott, 1977 and its associated microbial metagenome sequences

**DOI:** 10.12688/wellcomeopenres.24563.1

**Published:** 2025-07-23

**Authors:** Euichi Hirose, Jose Victor Lopez, Graeme Oatley, Elizabeth Sinclair, Eerik Aunin, Noah Gettle, Camilla Santos, Michael Paulini, Haoyu Niu, Victoria McKenna, Rebecca O’Brien, Wellcome Sanger Institute Tree of Life Management, Samples and Laboratory Team, Wellcome Sanger Institute Scientific Operations: Sequencing Operations, Wellcome Sanger Institute Tree of Life Core Informatics Team, EBI Aquatic Symbiosis Genomics Data Portal Team, Aquatic Symbiosis Genomics Project Leadership

**Affiliations:** 1 Faculty of Science, University of the Ryukyus, Nishihara, Okinawa, Japan; 2 National Coral Reef Institute, Department of Biological Sciences, Nova Southeastern University, Fort Lauderdale, Florida, USA; 3 Tree of Life, Wellcome Sanger Institute, Hinxton, England, UK

**Keywords:** Trididemnum clinides, cyanobacterial symbionts, genome sequence, photosymbiosis, chromosomal, Aplousobranchia

## Abstract

We present a genome assembly from a specimen of *Trididemnum clinides* (photosymbiotic ascidian; Chordata; Ascidiacea; Aplousobranchia; Didemnidae). The *T. clinides* genome sequence has a total length of 906.92 megabases. Most of the assembly (97.83%) is scaffolded into 23 chromosomal pseudomolecules. The mitochondrial genome has also been assembled and is 14.98 kilobases in length. The host ascidian has multiple symbionts, including *Prochloron*, a bacterial genus that can also synthesise bioactive natural products of interest for potential therapeutic development. Biosynthesis of active compounds sometimes involves microbial associates.

## Species taxonomy

Eukaryota; Opisthokonta; Metazoa; Eumetazoa; Bilateria; Deuterostomia; Chordata; Tunicata; Ascidiacea; Aplousobranchia; Didemnidae; *Trididemnum*; *Trididemnum clinides* Kott, 1977 (NCBI:txid641091)

## Background

In tropical and subtropical coral reef ecosystems, some colonial ascidians have evolved obligate symbiotic relationships with cyanobacteria, involving vertical transmission ( Hirose, 2015). This ascidian–cyanobacterial symbiosis is the only known life-long photosymbiosis among chordate taxa. These symbiotic systems have attracted the attention of many pharmaceutical scientists for their secondary metabolites ( Schmidt, 2015). The host ascidians are classified into four genera of the family Didemnidae (Tunicata: Ascidiacea: Aplousobranchia): *Didemnum*, *Trididemnum*, *Lissoclinum*, and *Diplosoma*. Among these, *Trididemnum* Della Valle, 1881 is distinguished by three rows of stigmata in the branchial sac and currently comprises 75 species ( Shenkar *et al.*, 2025). However, molecular phylogenetic analysis based on COI gene sequences has not yet confirmed the monophyly of this genus ( da Silva Oliveira *et al.*, 2017). At present, approximately ten *Trididemnum* species have been identified as photosymbiotic species.

*Trididemnum clinides* Kott, 1977 is a colonial ascidian recorded in Australia, French Polynesia, Eniwetak, Fiji, Guam, Philippines, Taiwan, Ryukyus (Okinawa), and Bonin Islands. Colonies harbor two types of unicellular cyanobacteria and one type of filamentous multicellular cyanobacteria in the cellulosic matrix of the host colony (i.e., tunic), whereas most photosymbiotic ascidian species harbour only one cyanobacterial species. The unicellular photosymbionts are regarded as *Prochloron* Lewin, 1977 and *Synechocystis* Sauvageau, 1892. However, the taxonomic affiliation of the filamentous cyanobacterium is uncertain. Few, if any *Prochloron* species have ever been continuously grown in laboratory cultures, despite intense interest from various angles – e.g. microbial, biochemical ( Lewin, 2012; Rumengan *et al*., 2020). Differences in the composition of photosynthetic pigments among the three suggested that each ‘‘species is distributed differently within the host tunic, depending on the light microenvironment ( Hirose *et al.*, 2009). The cushion-like colony is usually greyish in colour because of the dense distribution of calcareous spicules in the tunic. The spicules are less dense in shaded habitats, and the colony is often brownish because of cyanobacteria. While most symbiont cells are free from host cells and directly embedded in the matrix, some are engulfed by a tunic cell (haemocyte-like cells distributed in the tunic) and transferred to the host embryo brooded in the tunic ( Kojima & Hirose, 2012). In this vertical transmission process, host cells are unlikely to discriminate between the three symbiont species, and single tunic cell often carries multiple species of cyanobacteria.

Complex material exchanges may occur among the host and the three symbiotic cyanobacteria, and multiple cyanobacterial species may allow ascidian colonies to retain a variety of toxic compounds and use them for chemical defence. Various ascidian and tunicate species have been known to hold or generate bioactive metabolites. For example, antiviral activities were identified in a few Caribbean *Trididemnum* species ( Ramesh *et al.*, 2021)

Studying whole genome assembly of this species and its cyanobacterial symbionts may provide an understanding of the molecular mechanisms and evolution of this multi-photosymbiotic system, including interspecific interactions among the cyanobacterial symbionts in the ascidian host.

## Genome sequence report

### Sequencing data

**Figure 1.**
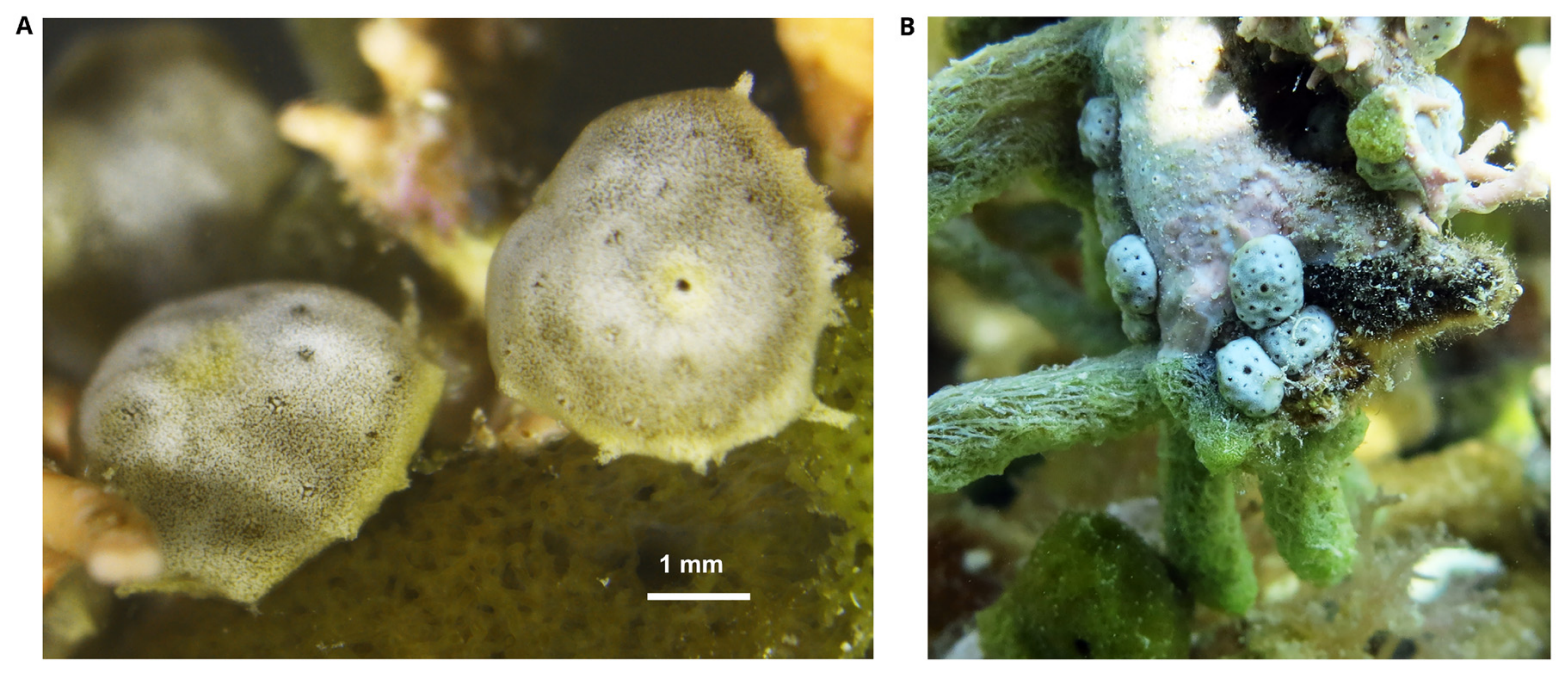
Photograph of the *Trididemnum clinides* (kaTriClin1) specimen used for genome sequencing. **A**. The specimen used for genome sequencing. **B**. *In situ* photograph of conspecific colonies neighboring the sample colony.

The genome of a specimen of *Trididemnum clinides* ( Figure 1) was sequenced using Pacific Biosciences single-molecule HiFi long reads, generating 30.91 Gb from 3.56 million reads. Based on the estimated genome size, the sequencing data provided approximately 27× coverage of the genome. Chromosome conformation Hi-C data produced 87.23 Gb from 577.69 million reads. RNA sequencing data were also generated and are available in public sequence repositories. Table 1 summarises the specimen and sequencing information.

**Table 1.** Specimen and sequencing data for *Trididemnum clinides*.

Project information			
**Study title**	Trididemnum clinides		
**Umbrella BioProject**	PRJEB66758		
**Species**	*Trididemnum clinides*		
**BioSpecimen**	SAMEA9873882		
**NCBI taxonomy ID**	641091		
Specimen information			
**Technology**	**ToLID**	**BioSample accession**	**Organism part**
**PacBio long read sequencing**	kaTriClin1	SAMEA9873897	Somatic tissue
**Hi-C sequencing**	kaTriClin1	SAMEA9873893	Somatic tissue
**RNA sequencing**	kaTriClin1	SAMEA9873895	Somatic tissue
Sequencing information			
**Platform**	**Run accession**	**Read count**	**Base count (Gb)**
**Hi-C Illumina NovaSeq 6000**	ERR12102423	5.78e+08	87.23
**PacBio Sequel IIe**	ERR12102457	5.97e+04	0.52
**PacBio Sequel IIe**	ERR12257376	2.24e+06	20.16
**PacBio Sequel IIe**	ERR12120136	1.26e+06	10.23
**RNA Illumina NovaSeq X**	ERR13093644	1.01e+07	1.53

### Assembly statistics

The primary haplotype was assembled, and contigs corresponding to an alternate haplotype were also deposited in INSDC databases. The assembly was improved by manual curation, which corrected 325 misjoins or missing joins and removed 123 haplotypic duplications. These interventions reduced the total assembly length by 5.12%, decreased the scaffold count by 20.33%, and also decreased the scaffold N50 by 6.45%. The final assembly has a total length of 906.92 Mb in 191 scaffolds, with 646 gaps, and a scaffold N50 of 38.01 Mb ( Table 2).

**Table 2.** Genome assembly data for *Trididemnum clinides*.

Genome assembly		
Assembly name	kaTriClin1.1	
Assembly accession	GCA_963675345.1	
*Alternate haplotype accession*	*GCA_963675475.1*	
Assembly level for primary assembly	chromosome	
Span (Mb)	906.92	
Number of contigs	837	
Number of scaffolds	191	
Longest scaffold (Mb)	62.08	
Assembly metric	Measure	*Benchmark*
Contig N50 length	2.29 Mb	*≥ 1 Mb*
Scaffold N50 length	38.01 Mb	*= chromosome N50*
Consensus quality (QV)	Primary: 60.3; alternate: 60.5; combined 60.4	*≥ 40*
*k*-mer completeness	Primary: 74.13%; alternate: 72.83%; combined: 93.58%	*≥ 95%*
BUSCO *	C:91.9%[S:88.2%,D:3.7%],F:2.9%,M:5.1%,n:954	*S > 90%, D < 5%*
Percentage of assembly assigned to chromosomes	97.84%	*≥ 90%*
Organelles	Mitochondrial genome: 14.98 kb	*complete single alleles*

* BUSCO scores based on the metazoa_odb10 BUSCO set using version 5.5.0. C = complete [S = single copy, D = duplicated], F = fragmented, M = missing, n = number of orthologues in comparison.

**Figure 2.**
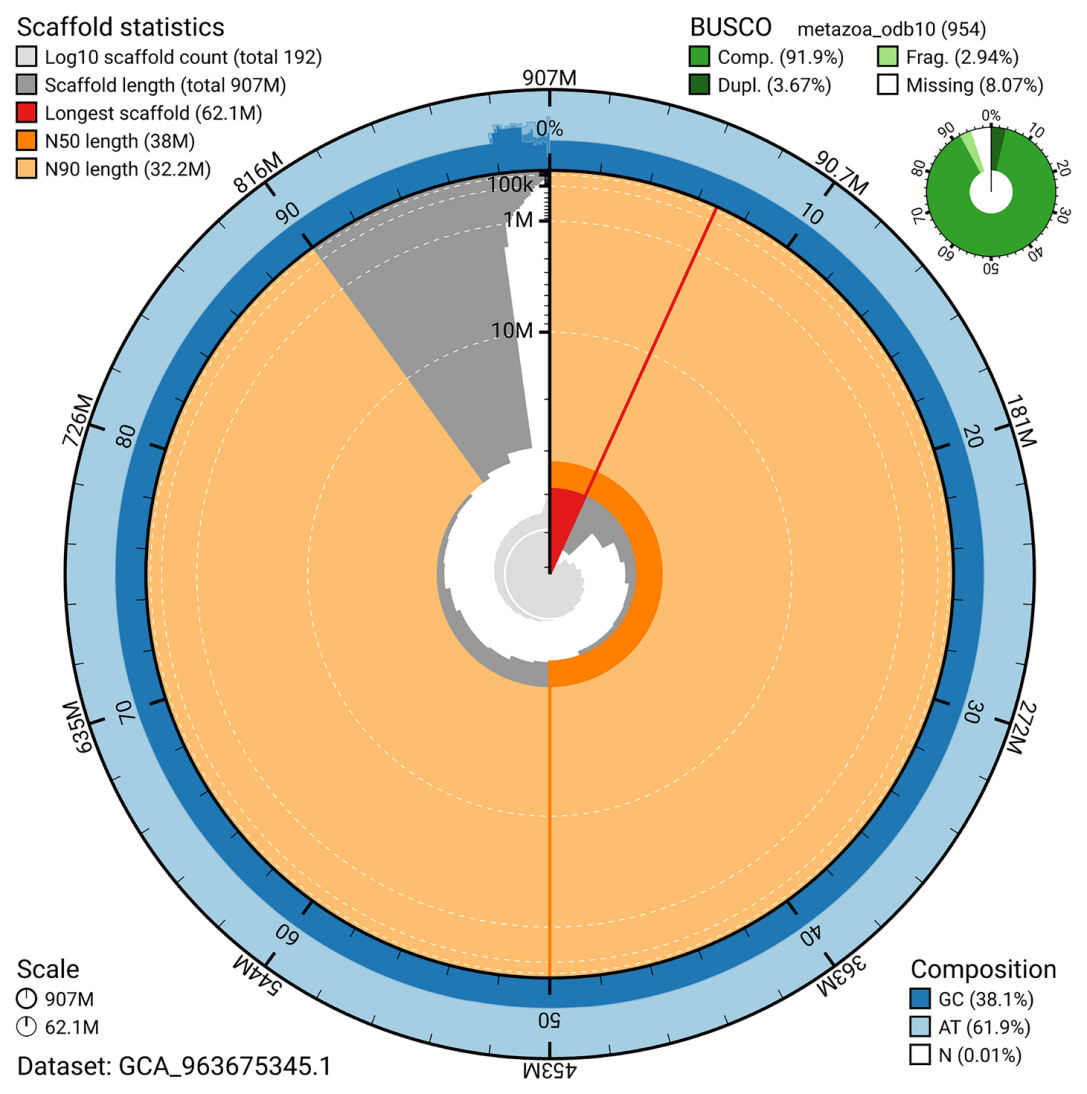
Genome assembly of *Trididemnum clinides*, kaTriClin1.1: metrics. The BlobToolKit snail plot provides an overview of assembly metrics and BUSCO gene completeness. The circumference represents the length of the whole genome sequence, and the main plot is divided into 1,000 bins around the circumference. The outermost blue tracks display the distribution of GC, AT, and N percentages across the bins. Scaffolds are arranged clockwise from longest to shortest and are depicted in dark grey. The longest scaffold is indicated by the red arc, and the deeper orange and pale orange arcs represent the N50 and N90 lengths. A light grey spiral at the centre shows the cumulative scaffold count on a logarithmic scale. A summary of complete, fragmented, duplicated, and missing BUSCO genes in the metazoa_odb10 set is presented at the top right. An interactive version of this figure is available at https://blobtoolkit.genomehubs.org/view/GCA_963675345.1/dataset/GCA_963675345.1/snail.

**Figure 3.**
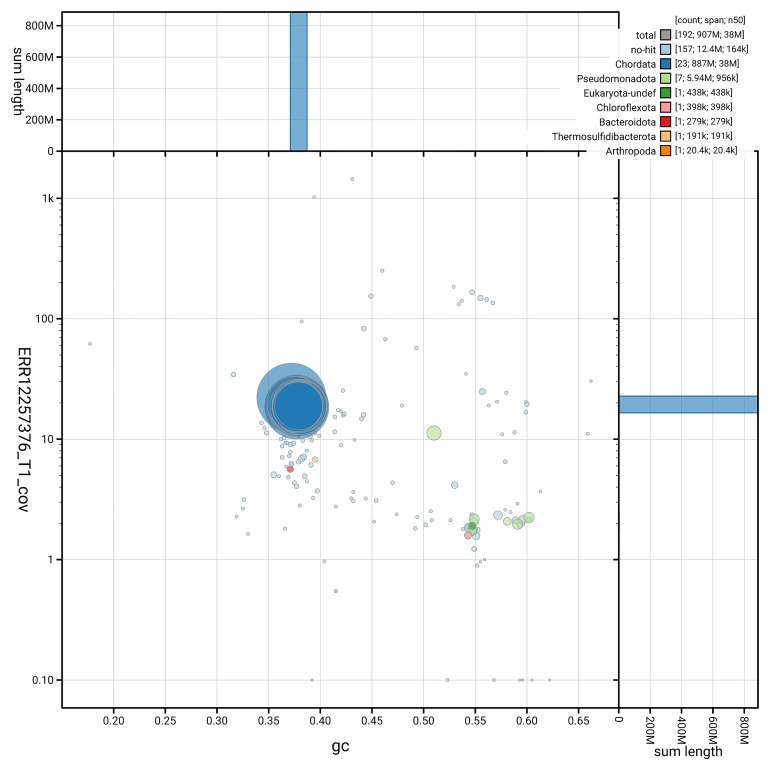
Genome assembly of *Trididemnum clinides*, kaTriClin1.1: BlobToolKit GC-coverage plot. Blob plot showing sequence coverage (vertical axis) and GC content (horizontal axis). The circles represent scaffolds, with the size proportional to scaffold length and the colour representing phylum membership. The histograms along the axes display the total length of sequences distributed across different levels of coverage and GC content. An interactive version of this figure is available at https://blobtoolkit.genomehubs.org/view/GCA_963675345.1/dataset/GCA_963675345.1/blob.

**Figure 4.**
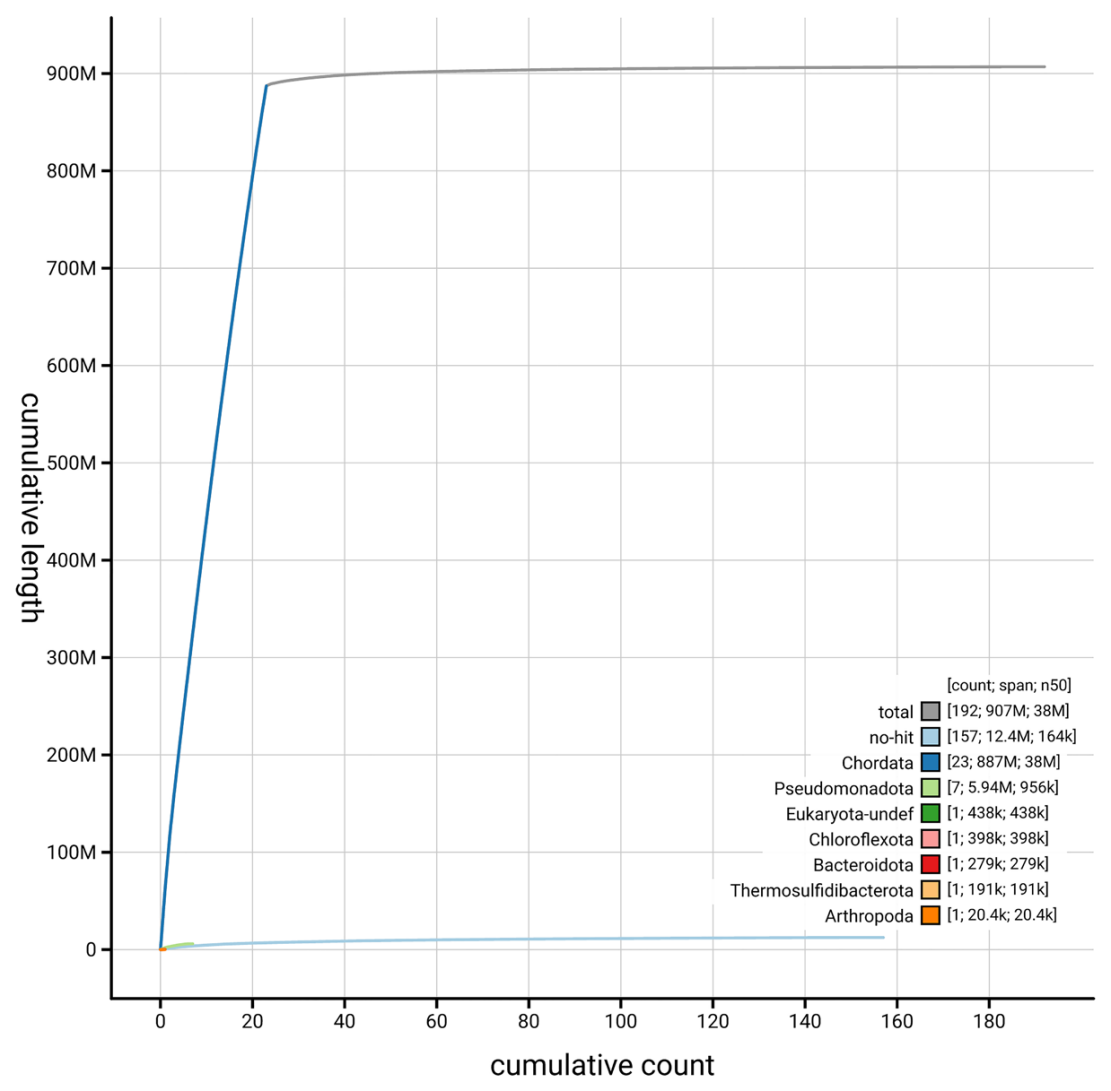
Genome assembly of *Trididemnum clinides,* kaTriClin1.1: BlobToolKit cumulative sequence plot. The grey line shows cumulative length for all scaffolds. Coloured lines show cumulative lengths of scaffolds assigned to each phylum using the buscogenes taxrule. An interactive version of this figure is available at https://blobtoolkit.genomehubs.org/view/GCA_963675345.1/dataset/GCA_963675345.1/cumulative.

The snail plot in Figure 2 provides a summary of the assembly statistics, indicating the distribution of scaffold lengths and other assembly metrics. Figure 3 shows the distribution of scaffolds by GC proportion and coverage. Figure 4 presents a cumulative assembly plot, with separate curves representing different scaffold subsets assigned to various phyla, illustrating the completeness of the assembly.

**Figure 5.**
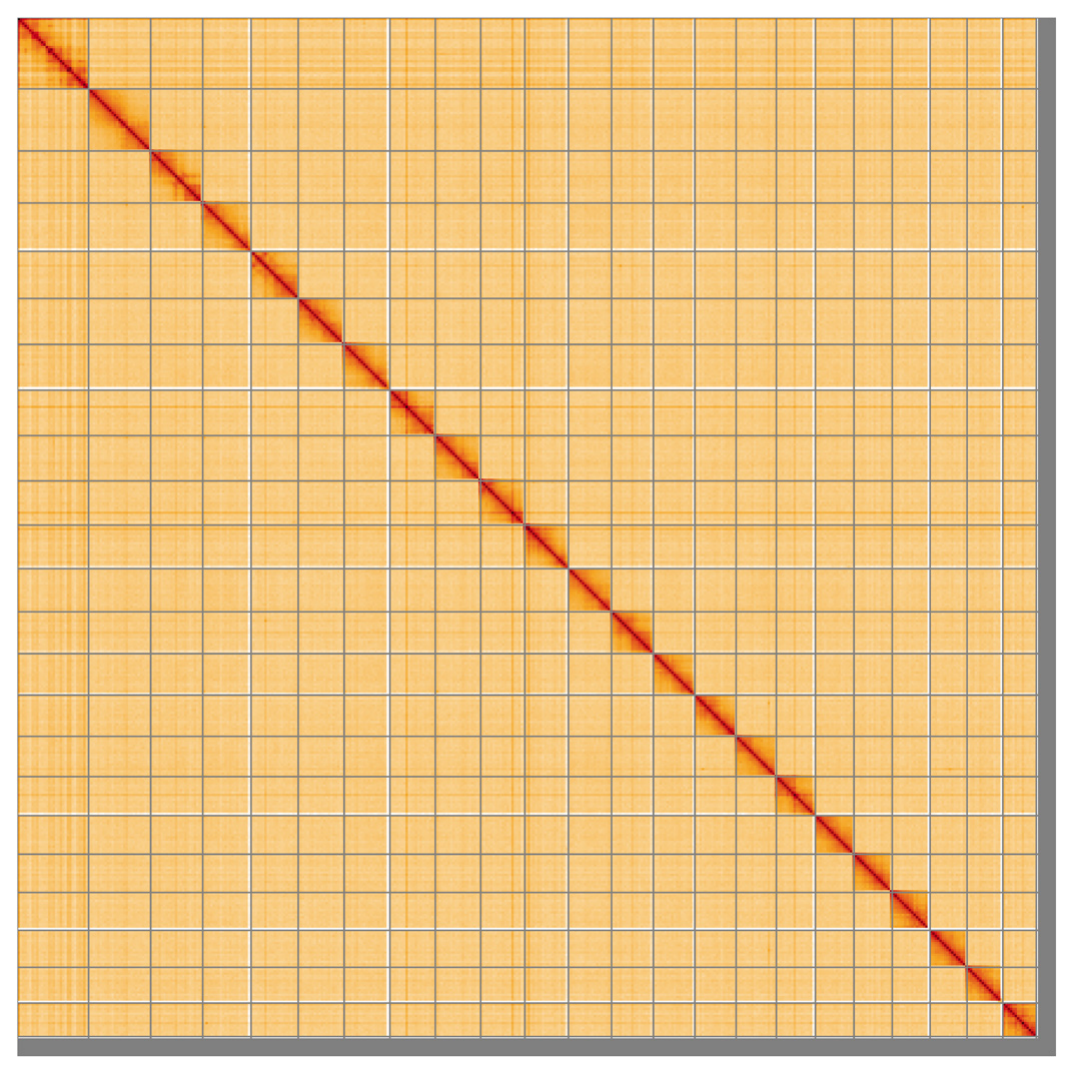
Genome assembly of *Trididemnum clinides*: Hi-C contact map of the kaTriClin1.1 assembly, visualised using HiGlass. Chromosomes are shown in order of size from left to right and top to bottom. An interactive version of this figure may be viewed at https://genome-note-higlass.tol.sanger.ac.uk/l/?d=E4TYsVOJT8yDJ9jWfP0xFA.

Most of the assembly sequence (97.84%) was assigned to 23 chromosomal-level scaffolds. These chromosome-level scaffolds, confirmed by Hi-C data, are named according to size ( Figure 5; Table 3).

**Table 3.** Chromosomal pseudomolecules in the genome assembly of *Trididemnum clinides*, kaTriClin1.

INSDC accession	Name	Length (Mb)	GC%
OY776284.1	1	62.08	37
OY776285.1	2	54.02	37.5
OY776286.1	3	45.32	38
OY776287.1	4	42.11	38
OY776288.1	5	41.04	38
OY776289.1	6	40.03	37.5
OY776290.1	7	39.83	38
OY776291.1	8	39.5	38
OY776292.1	9	39.46	38
OY776293.1	10	38.46	38
OY776294.1	11	38.01	37.5
OY776295.1	12	37.53	38
OY776296.1	13	36.46	38
OY776297.1	14	36.04	37.5
OY776298.1	15	36.0	38
OY776299.1	16	35.06	38
OY776300.1	17	33.93	38
OY776301.1	18	33.61	38
OY776302.1	19	33.31	38
OY776303.1	20	32.95	38
OY776304.1	21	32.23	38
OY776305.1	22	31.16	38
OY776306.1	23	29.14	38
OY776307.1	MT	0.01	17.5

The mitochondrial genome was also assembled. This sequence is included as a contig in the multifasta file of the genome submission and as a standalone record in GenBank.

### Assembly quality metrics

The estimated Quality Value (QV) and *k*-mer completeness metrics, along with BUSCO completeness scores, were calculated for each haplotype and the combined assembly. The QV reflects the base-level accuracy of the assembly, while *k*-mer completeness indicates the proportion of expected *k*-mers identified in the assembly. BUSCO scores provide a measure of completeness based on benchmarking universal single-copy orthologues.

The combined primary and alternate assemblies achieve an estimated QV of 60.4. The *k*-mer completeness is 74.13% for the primary haplotype and 72.83% for the alternate haplotype; and 93.58% for the combined primary and alternate assemblies. BUSCO v.5.5.0 analysis using the metazoa_odb10 reference set ( *n* = 954) identified 91.9% of the expected gene set (single =88.2%, duplicated =3.7%).

## Metagenome report

**Figure 6.**
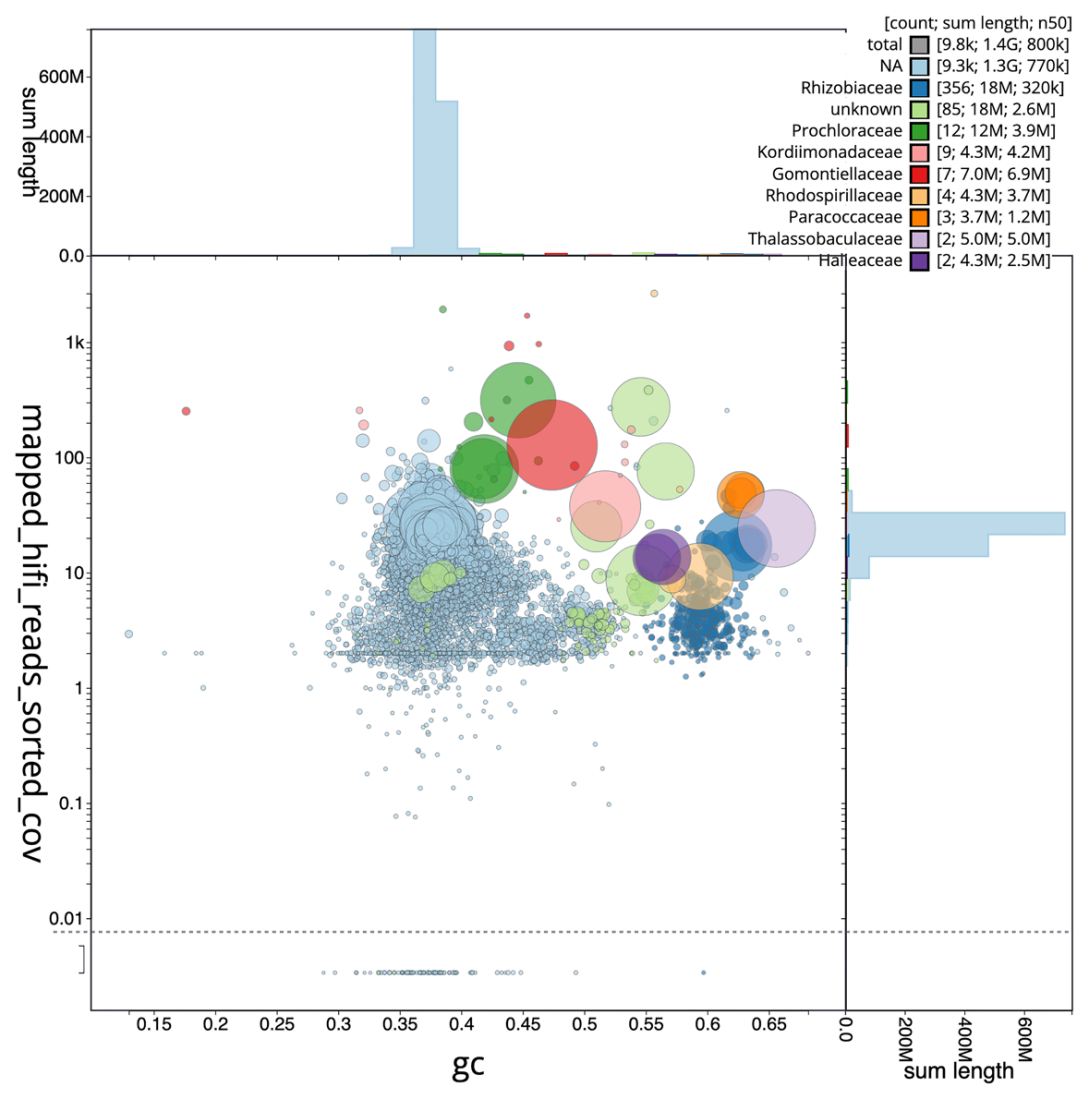
Blob plot of base coverage in mapped against GC proportion for sequences in the *Trididemnum clinides* metagenome. Binned contigs are coloured by family. Circles are sized in proportion to sequence length on a square-root scale, ranging from 510 to 6,879,066. Histograms show the distribution of sequence length sum along each axis.

**Figure 7.**
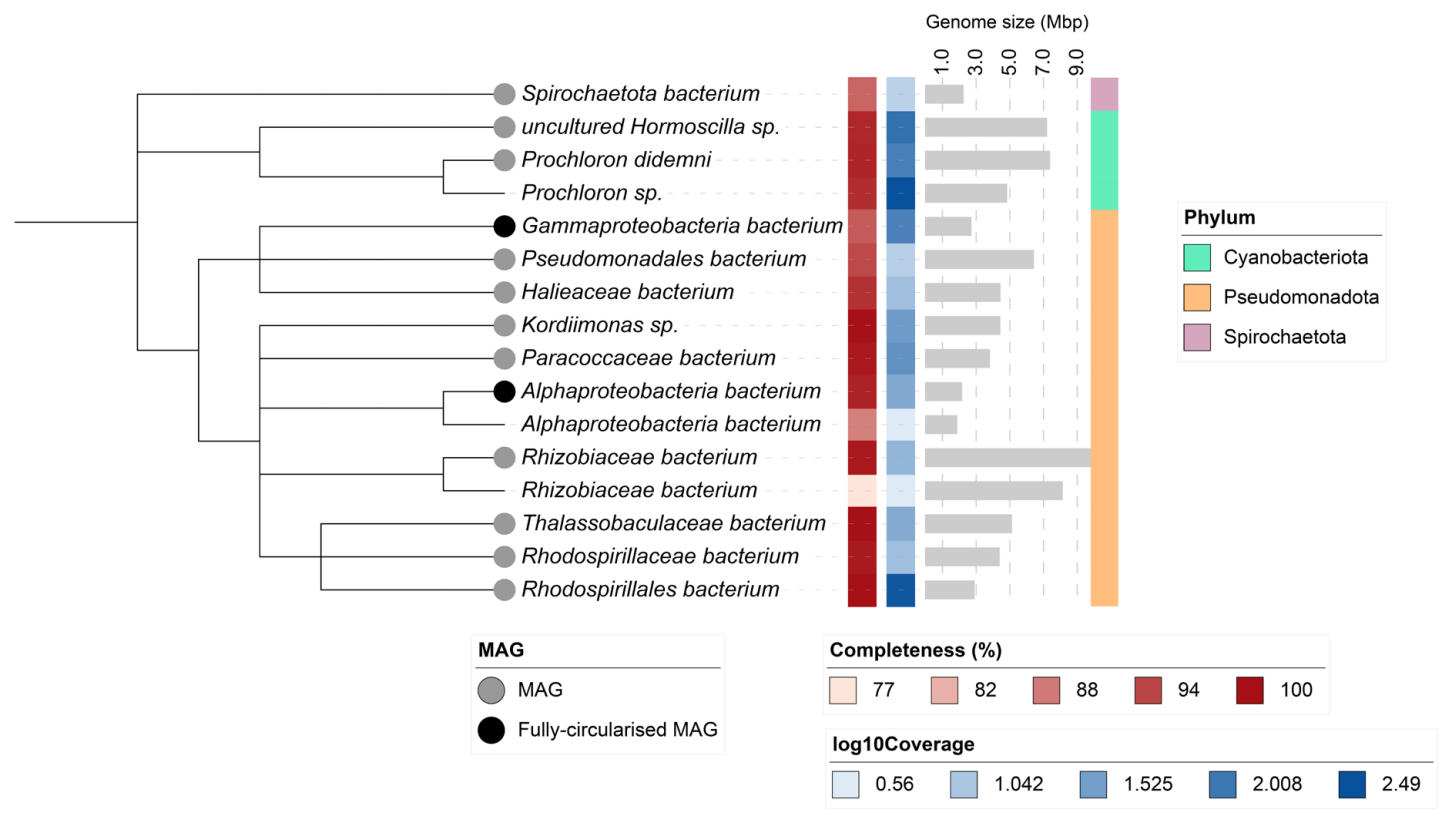
Cladogram showing the taxonomic placement of metagenome bins, constructed using NCBI taxonomic identifiers with taxonomizr and annotated in iTOL. Colours indicate phylum-level taxonomy. Additional tracks show sequencing coverage (log₁₀), genome size (Mbp), and completeness. Bins that meet the criteria for MAGs are marked with a grey circle; fully circularised MAGs are marked in black.

Sixteen binned genomes were generated from the metagenome assembly ( Figure 6), of which 13 were classified as high-quality metagenome assembled genomes (MAGs) (see methods). The completeness values for these assemblies range from approximately 76% to 100% with contamination below 5%. A cladogram of the binned metagenomes is shown in Figure 7. For details on binned genomes see Table 4.

**Table 4.** Quality metrics and taxonomic assignments of the binned metagenomes.

NCBI taxon	Taxid	GTDB taxonomy	Quality	Size (bp)	Contigs	Circular	Mean coverage	Completeness (%)	Contamination (%)
*Prochloron* sp.	1215	g__Prochloron	Medium	4,812,471	1	Yes	311.97	96.81	1.78
Rhizobiaceae bacterium	1913961	g__SPNT01	Medium	8,113,228	345	No	3.65	76.96	3.22
Alphaproteobacteria bacterium	1913988	o__UBA11136	Medium	1,851,293	47	Partial	3.69	87.96	1.8
uncultured *Hormoscilla* sp.	1007660	g__Hormoscilla	High	7,175,611	7	Partial	127.67	97.53	1.71
*Prochloron didemni*	1216	s__Prochloron didemni	High	7,355,447	11	Partial	83.48	97.7	0.67
Rhizobiaceae bacterium	1913961	g__SPNT01	High	9,807,038	11	Partial	17.4	99.22	2.25
Paracoccaceae bacterium	1904441	g__JANSYL01	High	3,785,794	3	No	47.64	99.09	0.45
Rhodospirillales bacterium	2026786	f__JAJFIE01	High	2,884,309	2	Partial	269.29	100	1.49
Rhodospirillaceae bacterium	1898112	f__Rhodospirillaceae	High	4,366,830	4	Partial	13.12	99	0.75
*Kordiimonas* sp.	1970157	g__Kordiimonas	High	4,405,740	9	Partial	37.69	99.91	2.17
Thalassobaculaceae bacterium	3014067	f__Thalassobaculaceae	High	5,098,954	2	Partial	23.87	99.78	1.3
Alphaproteobacteria bacterium	1913988	o__UBA11136	High	2,141,931	1	Yes	25.11	98.05	0
Halieaceae bacterium	2026743	f__Halieaceae	High	4,424,697	2	No	13.56	96.45	1.34
Pseudomonadales bacterium	1891229	f__HTCC2089	High	6,398,238	15	No	8.24	94.03	4.29
Gammaproteobacteria bacterium	1913989	f__UBA4486	High	2,686,766	1	Yes	75.44	92.02	1.94
Spirochaetota bacterium	2202144	f__COTS27	High	2,220,260	19	No	7.99	90.95	1.12

## Methods

### Sample acquisition

A *Trididemnum clinides* colony (specimen ID NSU00144301, ToLID kaTriClin1) was collected by snorkelling in the coral reef lagoon (<0.5 m in depth at low tide) of Bise, Okinawajima Island (Ryukyu Archipelago, Japan; latitude 26.71, longitude 127.88) on 2021-03-15. Colonies were attached to the basal portion of seagrass leaves ( *Thalassia hemprichii*) or dead coral branches. The colonies were preserved in DMSO–NaCl solution ( Dawson *et al.*, 1998). Taxonomic identification was performed following Kott (2001). Neighbouring conspecific colonies were donated to Ryukyu University Museum Fujukan (RUMF-ZU-00069).

### Nucleic acid extraction

The workflow for high molecular weight (HMW) DNA extraction at the Wellcome Sanger Institute (WSI) Tree of Life Core Laboratory includes a sequence of procedures: sample preparation and homogenisation, DNA extraction, fragmentation and purification. Detailed protocols are available on protocols.io ( Denton *et al.*, 2023b).

The kaTriClin1 sample was prepared for DNA extraction by weighing and dissecting it on dry ice ( Jay *et al.*, 2023). Tissue from the animal was homogenised using a PowerMasher II tissue disruptor ( Denton *et al.*, 2023a). HMW DNA was extracted using the Automated MagAttract v2 protocol ( Oatley *et al.*, 2023). DNA was sheared into an average fragment size of 12–20 kb in a Megaruptor 3 system ( Bates *et al.*, 2023). Sheared DNA was purified by solid-phase reversible immobilisation, using AMPure PB beads to eliminate shorter fragments and concentrate the DNA ( Strickland *et al.*, 2023). The concentration of the sheared and purified DNA was assessed using a Nanodrop spectrophotometer and Qubit Fluorometer using the Qubit dsDNA High Sensitivity Assay kit. Fragment size distribution was evaluated by running the sample on the FemtoPulse system.

RNA was extracted from tissue of kaTriClin1 in the Tree of Life Laboratory at the WSI using the RNA Extraction: Automated MagMax™ *mir*Vana protocol ( do Amaral *et al.*, 2023). The RNA concentration was assessed using a Nanodrop spectrophotometer and a Qubit Fluorometer using the Qubit RNA Broad-Range Assay kit. Analysis of the integrity of the RNA was done using the Agilent RNA 6000 Pico Kit and Eukaryotic Total RNA assay.

### Sequencing

Pacific Biosciences HiFi circular consensus DNA sequencing libraries were constructed according to the manufacturers’ instructions. DNA sequencing was performed by the Scientific Operations core at the WSI on Pacific Biosciences Sequel IIe. Tissue from the kaTriClin1 sample was processed for Hi-C sequencing at the WSI Scientific Operations core, using the Arima-HiC v2 kit and sequenced on the Illumina NovaSeq 6000 instrument. Poly(A) RNA-Seq libraries were constructed using the NEB Ultra II RNA Library Prep kit, following the manufacturer’s instructions. RNA sequencing was performed on the Illumina NovaSeq X instrument.

### Genome assembly, curation and evaluation

***Assembly***

Prior to assembly of the PacBio HiFi reads, a database of *k*-mer counts ( *k* = 31) was generated from the filtered reads using FastK. GenomeScope2 ( Ranallo-Benavidez *et al.*, 2020) was used to analyse the *k*-mer frequency distributions, providing estimates of genome size, heterozygosity, and repeat content.

The HiFi reads were assembled using Hifiasm ( Cheng *et al.*, 2021) with the --primary option. Haplotypic duplications were identified and removed using purge_dups ( Guan *et al.*, 2020). The Hi-C reads were mapped to the primary contigs using bwa-mem2 ( Vasimuddin *et al.*, 2019). The contigs were further scaffolded using the provided Hi-C data ( Rao *et al.*, 2014) in YaHS ( Zhou *et al.*, 2023) using the --break option for handling potential misassemblies. The scaffolded assemblies were evaluated using Gfastats ( Formenti *et al.*, 2022), BUSCO ( Manni *et al.*, 2021) and MERQURY.FK ( Rhie *et al.*, 2020).

The mitochondrial genome was assembled using MitoHiFi ( Uliano-Silva *et al.*, 2023), which runs MitoFinder ( Allio *et al.*, 2020) and uses these annotations to select the final mitochondrial contig and to ensure the general quality of the sequence.

***Assembly curation***

The assembly was decontaminated using the Assembly Screen for Cobionts and Contaminants (ASCC) pipeline. Flat files and maps used in curation were generated via the TreeVal pipeline ( Pointon *et al.*, 2023). Manual curation was conducted primarily in PretextView ( Harry, 2022) and HiGlass ( Kerpedjiev *et al.*, 2018), with additional insights provided by JBrowse2 ( Diesh *et al.*, 2023). Scaffolds were visually inspected and corrected as described by Howe *et al*. (2021). Any identified contamination, missed joins, and mis-joins were amended, and duplicate sequences were tagged and removed. The curation process is documented at https://gitlab.com/wtsi-grit/rapid-curation.

***Assembly quality assessment***

The Merqury.FK tool ( Rhie *et al.*, 2020), run in a Singularity container ( Kurtzer *et al.*, 2017), was used to evaluate *k*-mer completeness and assembly quality for the primary and alternate haplotypes using the *k*-mer databases ( *k* = 31) that were computed prior to genome assembly. The analysis outputs included assembly QV scores and completeness statistics.

A Hi-C contact map was produced for the final version of the assembly. The Hi-C reads were aligned using bwa-mem2 ( Vasimuddin *et al.*, 2019) and the alignment files were combined using SAMtools ( Danecek *et al.*, 2021). The Hi-C alignments were converted into a contact map using BEDTools ( Quinlan & Hall, 2010) and the Cooler tool suite ( Abdennur & Mirny, 2020). The contact map is visualised in HiGlass ( Kerpedjiev *et al.*, 2018).

The blobtoolkit pipeline is a Nextflow port of the previous Snakemake Blobtoolkit pipeline ( Challis *et al.*, 2020). It aligns the PacBio reads in SAMtools and minimap2 ( Li, 2018) and generates coverage tracks for regions of fixed size. In parallel, it queries the GoaT database ( Challis *et al.*, 2023) to identify all matching BUSCO lineages to run BUSCO ( Manni *et al.*, 2021). For the three domain-level BUSCO lineages, the pipeline aligns the BUSCO genes to the UniProt Reference Proteomes database ( Bateman *et al.*, 2023) with DIAMOND blastp ( Buchfink *et al.*, 2021). The genome is also divided into chunks according to the density of the BUSCO genes from the closest taxonomic lineage, and each chunk is aligned to the UniProt Reference Proteomes database using DIAMOND blastx. Genome sequences without a hit are chunked using seqtk and aligned to the NT database with blastn ( Altschul *et al.*, 1990). The blobtools suite combines all these outputs into a blobdir for visualisation.

The blobtoolkit pipeline was developed using nf-core tooling ( Ewels *et al.*, 2020) and MultiQC ( Ewels *et al.*, 2016), relying on the Conda package manager, the Bioconda initiative ( Grüning *et al.*, 2018), the Biocontainers infrastructure ( da Veiga Leprevost *et al.*, 2017), as well as the Docker ( Merkel, 2014) and Singularity ( Kurtzer *et al.*, 2017) containerisation solutions.

Table 5 contains a list of relevant software tool versions and sources.

**Table 5.** Software tools: versions and sources.

Software tool	Version	Source
BEDTools	2.30.0	https://github.com/arq5x/bedtools2
bin3C	0.3.3	https://github.com/cerebis/bin3C
BLAST	2.14.0	ftp://ftp.ncbi.nlm.nih.gov/blast/executables/blast+/
BlobToolKit	4.3.3	https://github.com/blobtoolkit/blobtoolkit
BUSCO	5.5.0	https://gitlab.com/ezlab/busco
bwa-mem2	2.2.1	https://github.com/bwa-mem2/bwa-mem2
CheckM	1.2.1	https://github.com/Ecogenomics/CheckM
Cooler	0.8.11	https://github.com/open2c/cooler
DAS Tool	-	https://github.com/cmks/DAS_Tool
DIAMOND	2.1.8	https://github.com/bbuchfink/diamond
dRep	3.4.0	https://github.com/MrOlm/drep
fasta_windows	0.2.4	https://github.com/tolkit/fasta_windows
FastK	427104ea91c78c3b8b8b49f1a7d6bbeaa869ba1c	https://github.com/thegenemyers/FASTK
Gfastats	1.3.6	https://github.com/vgl-hub/gfastats
GoaT CLI	0.2.5	https://github.com/genomehubs/goat-cli
GTDB-TK	2.3.2	https://github.com/Ecogenomics/GTDBTk
Hifiasm	0.19.5-r587	https://github.com/chhylp123/hifiasm
HiGlass	44086069ee7d4d3f6f3f0012569789ec138f42b84aa 44357826c0b6753eb28de	https://github.com/higlass/higlass
MaxBin	2.7	https://sourceforge.net/projects/maxbin/
MetaMDBG	1.1	https://github.com/GaetanBenoitDev/metaMDBG
MerquryFK	d00d98157618f4e8d1a9190026b19b471055b22e	https://github.com/thegenemyers/MERQURY.FK
MetaBat2	2.15-15-gd6ea400	https://bitbucket.org/berkeleylab/metabat/src/master/
MetaTOR	-	https://github.com/koszullab/metaTOR
Minimap2	2.24-r1122	https://github.com/lh3/minimap2
MitoHiFi	3	https://github.com/marcelauliano/MitoHiFi
MultiQC	1.14, 1.17, and 1.18	https://github.com/MultiQC/MultiQC
Nextflow	23.04.1	https://github.com/nextflow-io/nextflow
PretextView	0.2.5	https://github.com/sanger-tol/PretextView
PROKKA	1.14.5	https://github.com/vdejager/prokka
purge_dups	1.2.5	https://github.com/dfguan/purge_dups
samtools	1.18	https://github.com/samtools/samtools
sanger-tol/ascc	0.1.0	https://github.com/sanger-tol/ascc
sanger-tol/blobtoolkit	0.3.0	https://github.com/sanger-tol/blobtoolkit
Seqtk	1.3	https://github.com/lh3/seqtk
Singularity	3.9.0	https://github.com/sylabs/singularity
TreeVal	1.2.0	https://github.com/sanger-tol/treeval
YaHS	1.2a.2	https://github.com/c-zhou/yahs

### Metagenome assembly

The metagenome assembly was generated using MetaMDBG ( Benoit *et al.*, 2024) and binned using MetaBAT2 ( Kang *et al.*, 2019), MaxBin ( Wu *et al.*, 2014), bin3C ( DeMaere & Darling, 2019) and MetaTOR. The resulting bin sets of each binning algorithm were optimised and refined using Das Tool ( Sieber *et al.*, 2018). PROKKA ( Seemann, 2014) was used to identify tRNAs and rRNAs in each bin, CheckM ( Parks *et al.*, 2015) (checkM_DB release 2015-01-16) was used to assess bin completeness/contamination, and GTDB-TK ( Chaumeil *et al.*, 2022) (GTDB release 214) was used to taxonomically classify bins. Taxonomic replicate bins were identified using dRep ( Olm *et al.*, 2017) with default settings (95% ANI threshold). The final bin set was filtered for bacteria and archaea. All bins were assessed for quality and categorised as metagenome-assembled genomes (MAGs) if they met the following criteria: contamination ≤ 5%, presence of 5S, 16S, and 23S rRNA genes, at least 18 unique tRNAs, and either ≥ 90% completeness or ≥ 50% completeness with fully circularised chromosomes. Bins that did not meet these thresholds, or were identified as taxonomic replicates of MAGs, were retained as ‘binned metagenomes’ provided they had ≥ 50% completeness and ≤ 10% contamination. A cladogram based on NCBI taxonomic assignments was generated using the ‘taxonomizr’ package in R. The tree was visualised and annotated using iTOL ( Letunic & Bork, 2024). Software tool versions and sources are given in Table 5.

### Wellcome Sanger Institute – Legal and Governance

The materials that have contributed to this genome note have been supplied by a Tree of Life collaborator. The Wellcome Sanger Institute employs a process whereby due diligence is carried out proportionate to the nature of the materials themselves, and the circumstances under which they have been/are to be collected and provided for use. The purpose of this is to address and mitigate any potential legal and/or ethical implications of receipt and use of the materials as part of the research project, and to ensure that in doing so we align with best practice wherever possible. The overarching areas of consideration are:

•   Ethical review of provenance and sourcing of the material

•   Legality of collection, transfer and use (national and international)

Each transfer of samples is undertaken according to a Research Collaboration Agreement or Material Transfer Agreement entered into by the Tree of Life collaborator, Genome Research Limited (operating as the Wellcome Sanger Institute) and in some circumstances other Tree of Life collaborators.

## Data Availability

European Nucleotide Archive: Trididemnum clinides. Accession number PRJEB66758; https://identifiers.org/ena.embl/PRJEB66758. The genome sequence is released openly for reuse. The *Trididemnum clinides* genome sequencing initiative is part of the Aquatic Symbiosis Genomics (ASG) project ( https://www.ebi.ac.uk/ena/browser/view/PRJEB43743). All raw sequence data and the assembly have been deposited in INSDC databases. The genome will be annotated using available RNA-Seq data and presented through the Ensembl pipeline at the European Bioinformatics Institute. Raw data and assembly accession identifiers are reported in Table 1 and Table 2.

## Author information

Members of the Wellcome Sanger Institute Tree of Life Management, Samples and Laboratory Team are listed here: https://doi.org/10.5281/zenodo.13960213.

Members of the Wellcome Sanger Institute Scientific Operations: Sequencing Operations are listed here: https://doi.org/10.5281/zenodo.14870789.

Members of the Wellcome Sanger Institute Tree of Life Core Informatics team are listed here: https://doi.org/10.5281/zenodo.15495178.

Members of the European Bioinformatics Institute ASG Data Portal team are listed here: https://doi.org/10.5281/zenodo.10076466.

Members of the Wellcome Sanger Institute/Aquatic Symbiosis Genomics Project Leadership are listed here: https://doi.org/10.5281/zenodo.10184833.
