# Supplementary material for: The chromosomal genome sequence of the photosymbiotic ascidian, Trididemnum clinides Kott, 1977 and its associated microbial metagenome sequences
Source: Wellcome Open Res. 2025 Oct 6;10:357. Originally published 2025 Jul 23. [Version 2] doi: 10.12688/wellcomeopenres.24563.2 (PMC12432502; doi:10.12688/wellcomeopenres.24563.2)
Supplement: Peer review history

# Reviewer report: Reviewer response for version 1

**DOI:** 10.21956/wellcomeopenres.27072.r128350

**Published:** 2025-09-12

**Authors:** Kevin M. Kocot

**Affiliations:** 1 Department of Biological Sciences,, University of Alabama,, Tuscaloosa,, AL,, USA

In this manuscript, Hirose et al. describe the chromosomal genome assembly of a photosymbiotic ascidian and the genomes of associated prokaryotes. I found all aspects of the manuscript to be thorough and appropriately detailed. The manuscript is generally very well-written. Overall, I think the manuscript is suitable for publication as-is but see a few minor comments below:

 •    Abstract: If space permits, it might be worthwhile to clarify that Prochloron is a cyanobacterial symbiont.

 •    In the Keywords and Data availability sections, Trididemnum clinides should be italicized.

 •    Background: There is a grammatical issue (I think a word is missing) in the first sentence of the last paragraph of this section.

 •    Sequencing data section of Genome sequence report section: There is an unnecessary hyphen in “con-formation” in the second-to-last paragraph of this section.

 •    Table 1: Could any more specific details be provided about what tissue(s)/material were used? Was any tunic material included? If the gut was intentionally excluded, that might be valuable to note.

 •    Methods: Please note the storage temperature of the tissues stored in DMSO-NaCl buffer and approximately how long it was stored prior to nucleic acid extraction. Please also clarify that the tissue used for RNA extraction was also preserved in this manner.

 •    Figure 7: Words like “uncultured” and “sp.” do not need to be italicized.

Are sufficient details of methods and materials provided to allow replication by others?

Yes

Is the rationale for creating the dataset(s) clearly described?

Yes

Are the datasets clearly presented in a useable and accessible format?

Yes

Are the protocols appropriate and is the work technically sound?

No

Reviewer Expertise:

NA

I confirm that I have read this submission and believe that I have an appropriate level of expertise to confirm that it is of an acceptable scientific standard.

# Reviewer report: Reviewer response for version 1

**DOI:** 10.21956/wellcomeopenres.27072.r128359

**Published:** 2025-08-30

**Authors:** Xavier Turon

**Affiliations:** 1 Department of Marine Ecology, Centre d’Estudis Avançats de Blanes (CEAB-CSIC), Blanes,, Spain

I have read this article with interest and, in my view, it provides new and relevant information. The format is the same I see in other Wellcome Open Research papers, as are the protocols followed, graphs, and tables. So, I imagine these are standard for this type of publication and I am not arguing with them.

 The parameters of the assembly of the genome and associated metagenomes are correct.

 I want to emphasize that T. clinides is a well-known model, examined from morphological and biological points of view previously by the first author.

 What I miss (but I don’t know if it falls in the scope of this type of reports) is a link between previous observations and the metagenome results. The species is known to harbor three main cyanobacterial species, likely a Prochloron, a Synechocystis, and a filamentous cyanobacterium. How do this relate to the 16 binned metagenomes? Do they confirm the identity of the vertically transmitted symbionts? I see no Synechocystis among the MAGs, is the Hormoscilla consistent with the filamentous unidentified symbiont?

 I would also have appreciated more comments, at least on how the chromosomal number inferred related to that found in other didemnids, or about the mitochondrial genome (number of genes, comparison with closely related groups…). But, as said, I infer that this is the format of this type of notes, and that this information can be further elaborated in future works.

 Picture 1A does not seem an in situ image, please

 clarify in legend if it has been taken at the lab.

 Typos:

 “Few, if any Prochloron species have ever been continuously…” should be “Few, if any, Prochloron...”

 Close quotation mark in: Differences in the composition of photosynthetic pigments among the three suggested that each ‘‘species is distributed differently”

 Correct: Avail-able in public sequence repositories

Are sufficient details of methods and materials provided to allow replication by others?

Yes

Is the rationale for creating the dataset(s) clearly described?

Yes

Are the datasets clearly presented in a useable and accessible format?

Yes

Are the protocols appropriate and is the work technically sound?

Yes

Reviewer Expertise:

Marine Biology, Ascidians

I confirm that I have read this submission and believe that I have an appropriate level of expertise to confirm that it is of an acceptable scientific standard.
